# Predicting sequence and structural specificities of RNA binding regions recognized by splicing factor SRSF1

**DOI:** 10.1186/1471-2164-12-S5-S8

**Published:** 2011-12-23

**Authors:** Xin Wang, Liran Juan, Junjie Lv, Kejun Wang, Jeremy R Sanford, Yunlong Liu

**Affiliations:** 1 Center for Computational Biology and Bioinformatics, Indiana University School of Medicine, IN 46202, USA; 2 College of Automation, Harbin Engineering University, Harbin, Heilongjiang 150001, China; 3 Department of Medical and Molecular Genetics, Indiana University School of Medicine, IN 46202, USA; 4 Department of Molecular, Cellular and Developmental Biology, University of California Santa Cruz, Santa Cruz, California 95064, USA; 5 Center for Medical Genomics, Indiana University School of Medicine, Indianapolis, IN 46202, USA

## Abstract

**Background:**

RNA-binding proteins (RBPs) play diverse roles in eukaryotic RNA processing. Despite their pervasive functions in coding and noncoding RNA biogenesis and regulation, elucidating the sequence specificities that define protein-RNA interactions remains a major challenge. Recently, CLIP-seq (Cross-linking immunoprecipitation followed by high-throughput sequencing) has been successfully implemented to study the transcriptome-wide binding patterns of SRSF1, PTBP1, NOVA and fox2 proteins. These studies either adopted traditional methods like Multiple EM for Motif Elicitation (MEME) to discover the sequence consensus of RBP's binding sites or used Z-score statistics to search for the overrepresented nucleotides of a certain size. We argue that most of these methods are not well-suited for RNA motif identification, as they are unable to incorporate the RNA structural context of protein-RNA interactions, which may affect to binding specificity. Here, we describe a novel model-based approach--*RNAMotifModeler *to identify the consensus of protein-RNA binding regions by integrating sequence features and RNA secondary structures.

**Results:**

As an example, we implemented *RNAMotifModeler *on SRSF1 (SF2/ASF) CLIP-seq data. The sequence-structural consensus we identified is a purine-rich octamer 'AGAAGAAG' in a highly single-stranded RNA context. The unpaired probabilities, the probabilities of not forming pairs, are significantly higher than negative controls and the flanking sequence surrounding the binding site, indicating that SRSF1 proteins tend to bind on single-stranded RNA. Further statistical evaluations revealed that the second and fifth bases of SRSF1octamer motif have much stronger sequence specificities, but weaker single-strandedness, while the third, fourth, sixth and seventh bases are far more likely to be single-stranded, but have more degenerate sequence specificities. Therefore, we hypothesize that nucleotide specificity and secondary structure play complementary roles during binding site recognition by SRSF1.

**Conclusion:**

In this study, we presented a computational model to predict the sequence consensus and optimal RNA secondary structure for protein-RNA binding regions. The successful implementation on SRSF1 CLIP-seq data demonstrates great potential to improve our understanding on the binding specificity of RNA binding proteins.

## Introduction

RNA-binding proteins (RBPs) are implicated in virtually every step of post-transcriptional gene expression including pre-mRNA splicing, RNA editing and polyadenylation [1]. These proteins possess a diverse array of structurally and functionally distinct RNA-binding domains such as RNA recognition motifs (RRM), KH domains, RGG boxes, zinc finger, double-stranded RNA-binding domain, etc [1]. In contrast to DNA, recognition sites for RNA binding proteins can be presented diverse structural contexts. Indeed the structural context of binding sites can have pronounced effects on protein-RNA interactions [2,3]. Likewise, RNA binding proteins can alter the folding landscape of RNA molecules thereby inducing structured or single stranded conformations [4]. Given the significant role RNA folding plays in promoting or inhibiting protein-RNA interactions methods for evaluating both the sequence and RNA-structural determinants to binding specificity will be highly beneficial to the field.

Several methods for elucidating the specificity of protein-RNA interactions enable rapid advances in our understanding of RBP functions. One recent innovation is the Cross-Linking ImmunoPrecipitation (CLIP); CLIP exploits photoreactive residues in RNA and polypeptides to generate covalently linked complexes. Because UV irradiation does not induce protein-protein cross-links CLIP is thought to be more specific than other IP based assays for protein-RNA interactions. CLIP was successfully applied to identify mRNA targets of the NOVA protein, a neural splicing factor associated with paraneoplasticopsoclonus myoclonus ataxia (POMA) [5-7]. Coupling CLIP with next-generation high-throughput sequencing technology, known as CLIP-seq or HITS-CLIP, provides a cost-efficient method to increase the sensitivity of the assay by surveying the RNA landscape on a more global scale. Several groups have successfully implemented CLIP-seq analysis of NOVA, SRSF1, fox2 and PTB proteins in mammalian systems [5,8-10]. Both MEME and Z-score statistics have been used to reveal consensus binding motifs that are overrepresented in CLIP-seq data [5,9]. Although Z-score statistics may be able to identify the overrepresented sequence motifs, it does not consider the degenerate feature of the binding specificities of RBPs. MEME-based method is well known to be an excellent tool for cases only regarding sequence specificity [11]. Neither of these approaches canascertain the roles of RNA secondary structure in establishing the context of the protein-RNA interaction. Hiller et al. extended MEME by adding a pre-computing procedure to measure single-strandedness of RNA sequence as *a prior *knowledge to guide the motif search. They demonstrated that their model, MEMERIS, is able to identify binding motifs located in single-stranded regions with both artificial and biological data [12]. Recently, Kazan et al. proposed *RNAcontext *for learning both sequence and structural binding preferences of RNA-binding proteins [13].

Here we describe a model-based approach--*RNAMotifModeler *to evaluate protein-RNA interactionsusinga retained binding affinity ratio, which is considered to be affected by two major factors--sequence degeneracy and RNA secondary structure deviation. *RNAMotifModeler *incorporates predicted unpaired probability of each nucleotide in the protein-RNA binding regions; such probability is derived from RNA secondary prediction algorithms, such as RNA-fold, based on the nucleotide compositions of the neighbouring flanking sequences. This strategy is different from *RNAContext*, which uses predicted RNA secondary structures as input such as 'Paired', 'Hairpin Loop', 'Unstructured' or 'Miscellaneous'. Unlike *MEMERIS*, *RNAMotifModeler *uses the base-pairing probability for each nucleotide rather than the entire binding site. For each binding instance, *RNAMotifModeler *defines a score that evaluates the consensus binding site within an optimal structural context, and aims at searching for an optimal RNA sequence-structural consensus for an RNA binding protein. These features enhance our ability to calculate and estimate the sequences that yield the highest binding affinity for a specific RBP.

We tested *RNAMotifModeler *on CLIP-seq data that profile the transcriptome-wide binding pattern of SRSF1, serine/arginine-rich splicing factor 1 [5]. The sequence features of the binding motifs are consistent with the experimentally defined *cis*-acting elements recognized by SRSF1 [5,14,15]. Interestingly, the prediction suggests that the second and fifth bases of SRSF1octamer motif have stronger sequence specificities, but lower p-values of unpaired probabilities, while the third, fourth, sixth and seventh bases are more significantly to be single-stranded, but have less sequence specificities. Therefore, we conclude that the sequence and structure specificities are both required and may play complementary roles during binding site recognition of SRSF1.

## Results

Elucidating the sequence and structural features defining protein-RNA interactions is a major challenge in the field. To begin to address this problem we developed a tool to evaluate the structural context of RNA fragments co-purified with RNA binding proteins by CLIP. The results presented here focus on SRSF1; however this tool will be generally applicable to any RNA binding protein. SRSF1 is an essential splicing factor with multiple roles in post-transcriptional gene expression [16]. SRSF1 is also a potent proto-oncogene and implicated in maintaining genome stability [17]. Moreover, loss of SRSF1 binding sites by mutations linked to genetic diseases can induce aberrant patterns of pre-mRNA splicing [5]. Thus considerable effort has been focused on defining the binding specificity and RNA targets of SRSF1. Here we report a novel tool intended to examine the contributions of structural and sequence elements in RNA fragments co-purified with SRSF1 by CLIP.

### Workflow of RNAMotifModeler

The first step of RNAMotifModeler is to do data preprocessing. In this study, the data came from our previous genome-wide profiling of SRSF1 protein's binding sites by combining cross-linking immunoprecipitation (CLIP) with high-throughput sequencing [5]. In total, 932,152 reads were obtained from SFRS1-bound RNA in four independent experiments. As a comparison, 670,448 reads were generated from three experiments performed on nonselected input RNA. After removing redundant sequences and alignment to the human genome, we obtained 953 and3374 loci for CLIP and input RNA samples, respectively.904 positive gold standard sequences were selected from at least three out of the four CLIP-seq experiments and absent from the input sequences. A same number of negative sequences were randomly picked from non-SRSF1-targeted regions belonging to the same genomic category (exonic, intronic, intergenic, etc) as their positive counterparts. Basepairing probabilities of each nucleotide to its neighbours were subsequently predicted by *RNAfold *[18] (ViennaRNA package, version 1.7.2) for both positive and negative gold standard sequences.

**Figure 1:**
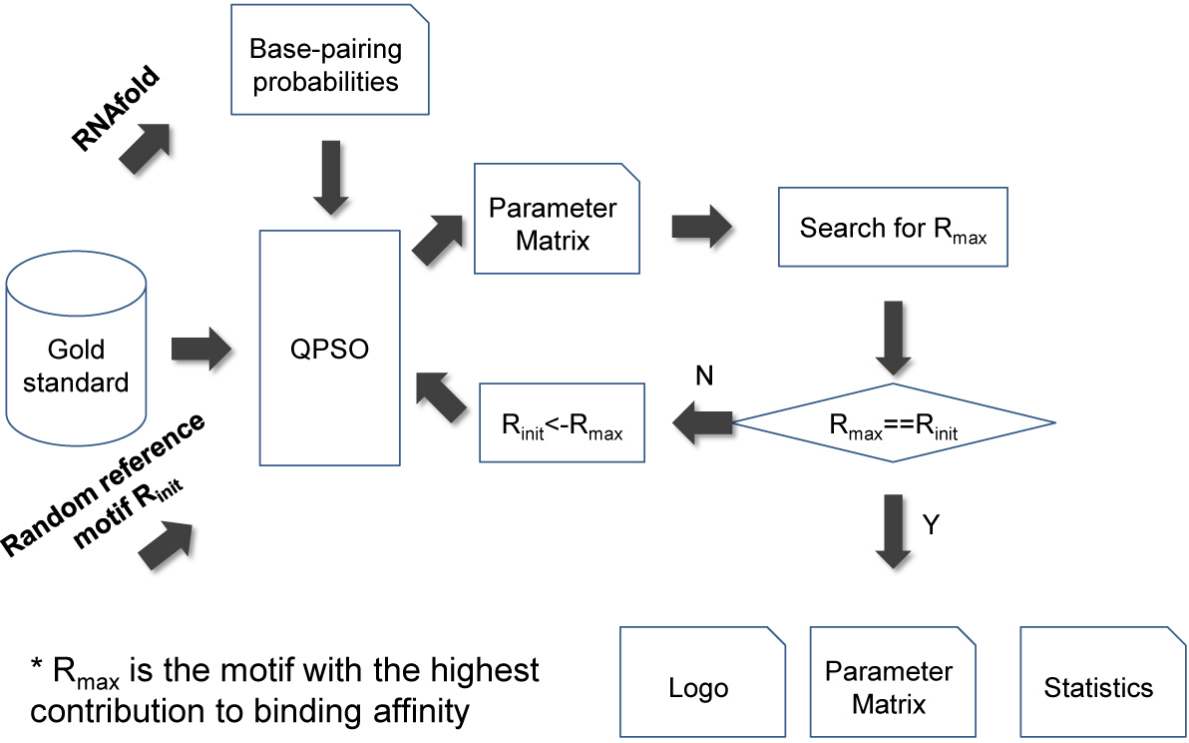
**Workflow of RNAMotifModeler**.

Our next step, as shown in Figure 1, is to identify sequence-structural consensus for the RNA binding protein based on the gold standard sequences from CLIP-seq data. We took an iterative approach that alternates between: 1) optimization of parameters specifying sequence degeneracy and structural context given a sequence motif, and 2) search of optimal sequence reference motif given the estimated parameters by evaluation of each motif candidate's contribution to binding affinities of positive gold standard sequences. The above two steps will be repeated until a convergence when the starting motif candidate makes the most contribution to binding affinities.

Finally, RNAMotifModeler outputs the converged sequence motif, optimal parameters, statistical performance of using the optimal parameters such as the area under the ROC curve (AUC), etc. The AUC scores are measured by area under ROC curves derived from predictions of gold standard sequences being bound by SRSF1 proteins varying the binding affinity threshold. In order to predict binding sites of SRSF1 proteins, we pick the sequence binding affinity yielding the maximal prediction accuracy as a cutoff score. Based on the predicted parameters, positive gold-standard sequences can be scanned to find all potential binding sites with binding affinities higher than the cutoff score. These binding sites can be further used for sequence logo creation and transformed to positional weight matrix, which aremuch more widely used.

### Convergence of SRSF1 consensus motif searching

**Figure 2:**
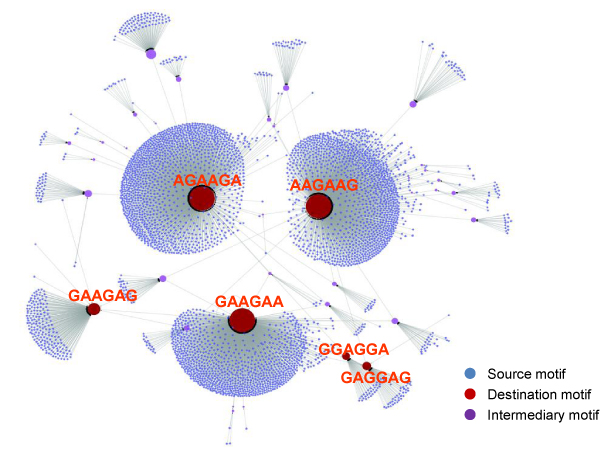
**Motif searching graph**. Source, intermediary and destination motifs are represented by nodes colored in blue, purple and red. The size of node is proportional to its in-degree. Arrows between nodes indicate reference motif searching pathways. This figure demonstrates the fast convergence of the vast majority of motif candidates using the Quantum Particle Swarm Optimization algorithm.

We call the converging path from a starting motif candidate to the final consensus motif a *motif searching pathway*. To have a global overview of the convergence, motif searching pathways for all motif candidates are organised together to form a *motif searching graph*. In the particular case of hexamer prediction for SRSF1, the motif searching pathwaysof all initialized reference motifs converge to a short list of candidates (Figure 2). All of the 4096 motif candidates converge within three iterations, of which 85.7% converge after the first iteration. AGAAGA, AAGAAG and GAAGAA are top three hexamers with the highest in-degrees, responsible for 99.7% of all motif candidates (Table 1). Despite only one or two sequence alterations, the other twelvereference motifs are closely related to these three motifs. It is also notedthat nearly an equal number of motif candidates convergeto each one of the top three reference motifs. More interestingly, these hexamers share a core sequence of 'AAGA'indicating that they may be adjacent to each other in RNA fragments.

**Table 1:** The final converged motifs and their corresponding numbers of source motifs

Converged motif	No. of source motifs
AGAAGA	1484
AAGAAG	1375
GAAGAA	1225
Others	12

### SRSF1 consensus motifs of different lengths

RNAMotifModeler provides an option to predict sequence-structural consensus of different lengths. We have mentioned in the previous section that for short motifs, it is suggested to perform predictions starting from every potential motif candidate and generate a motif searching graph to inspect the global convergence. For longer motifs, however, it is computationally expensive. In this case, we conduct predictions starting froma sufficient number of motif candidates randomly picked from motif space. The converged motif with the highest prediction power, measured by AUC, is selected as the optimal one.

Using the above strategy, we predicted optimal 6nt, 7nt and 8nt consensus motifs for SRSF1 proteins (Additional file 1(A), (B) and Table 2). Interestingly, the sequence motifs of different lengths are highly similar to each other. Comparing their sequence and structural parameters identified, we can also see a high consistency among them. Importantly, the predicted unpaired probabilities of these three motifs indicate SRSF1 tends to bind on single-stranded RNA regions.

**Table 2:** Optimal parameters of the octamer predicted by RNAMotifModeler.

	A	G	A	A	G	A	A	G
**A**	1.00	0.17	1.00	1.00	0.24	1.00	1.00	0.81
**G**	0.79	1.00	0.65	0.90	1.00	0.84	1.00	1.00
**C**	0.52	0.32	0.50	0.16	0.35	0.02	0.34	0.63
**U**	0.75	0.15	0.39	0.63	0.09	0.06	0.73	0.55
**UP**	0.99	0.96	0.99	0.99	0.98	0.99	0.92	0.83

The column headers represent the predicted reference motif. The first four rows indicate the retained binding affinity ratio corresponding to a sequence alteration from each nt in the reference motif. The last row shows the optimal unpaired probability (UP) for each nt of the predicted reference motif.

### Predicted sequence and structural features of SRSF1 binding regions

**Figure 3:**
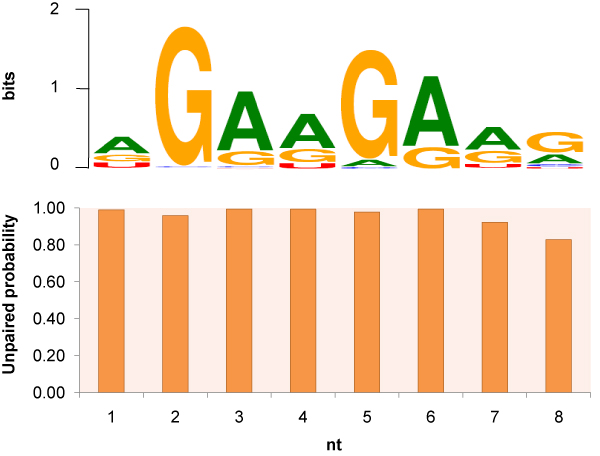
**Sequence consensus logo (the upper panel) and unpaired probabilities (the lower panel) of SRSF1 predicted by RNAMotifModeler**.

**Figure 4:**
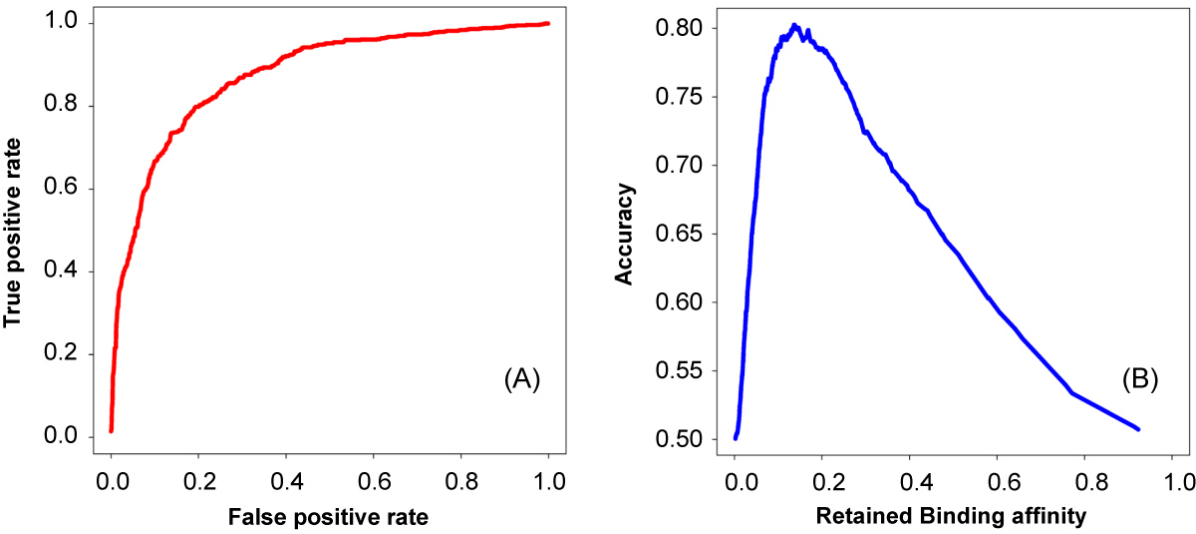
**ROC curve and accuracy curve describing the prediction power of RNAMotifModeler for SRSF1 proteins**.

To better compare RNAMoifModeler predictions with the SRSF1 binding motif reported previously, here we focus on octamer predictions. Consistent with the sequence consensus predicted by MEME [5], the reference sequence motif for SRSF1 proteins predicted using RNAMotifModeler is also 'AGAAGAAG' (Table 2 and Figure 3). Based on the predicted optimal parameters, we obtained an AUC of 0.875 (Figure 4(A)) and an maximal accuracy of 0.803 (Figure 4(B)), which are both higher than the MEME-based prediction, of which the AUC is 0.86 and maximal accuracy is 0.78 [5]. The optimal parameter matrix searched by RNAMotifModeler is presented in Table 2. The first row listed the reference sequence motif identified while the following four rows include retained binding affinity ratios caused by sequence alterations. To visualize the predicted SRSF1 sequence consensus more straightforwardly, positive gold-standard sequences were scanned to search binding sites with binding affinities higher than the threshold 0.138, based on which a sequence logo was created by Weblogo [19]. This motif is consistent with the positional weight matrix (PWM) identified by MEME using the same gold standard sequences in our previous study [5], and is similar to the motifs found by other groups [20-22]. The last row in Table 2 is constituted by unpaired probabilities for all nucleotides in the motif, indicating the optimal RNA secondary structure of SRSF1 binding regions. We note that every nucleotide of the predicted SRSF1 binding motif has a very high probability to be single-stranded, suggesting that SRSF1 proteins tend to bind on highly unpaired RNA regions.

### SRSF1-RNA binding sites are presented as single-stranded regions

**Figure 5:**
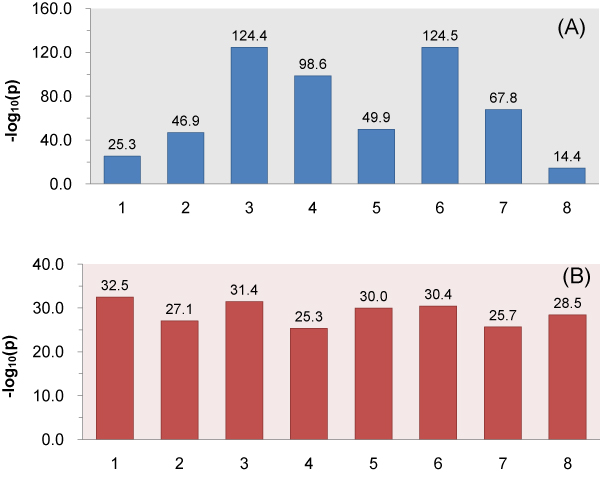
**Results of Wilcoxon tests with the alternative hypothesis thatthe unpaired probabilities in the predicted binding sites are higher than negative controls randomly drawn from (A) the same positive gold-standard sequences, and (B) random sequences**.

**Figure 6:**
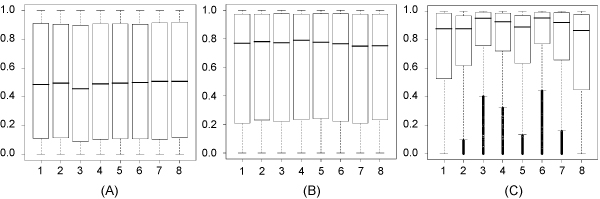
**Unpaired probabilities of nucleotides of (A) a control set of binding sites randomly selected from negative goldstandard sequences (B) a control set of binding sites randomly selected from positive goldstandard sequences, and (C) binding sites predicted by RNAMotifModeler in positive gold standard sequences**.

Based on the predicted unpaired probabilities of nucleotides in the converged reference motif, we can conclude that SRSF1 tends to recognize purine-rich motifs in regions with a low degree of predicted secondary structure. To further test the hypothesis that RNA regions bound by SRSF1 proteins are significantly unpaired, we compared the unpaired probability of each nucleotide in the predicted binding sites with two different control sets of random'binding'sites. In our first control set, we randomly picked the same number of sites as the predicted binding sites in each positive gold standard sequence. P-values were computedfor each nucleotide based on Wilcoxon rank sum tests, with the alternative hypothesis that the unpaired probability in predicted binding sites are higher than controls. Indeed, all median unpaired probabilities of predicted binding sites are significantly higher than controls (Figure 5(A)). In the second control set, we first randomly selected the same number of exonic fragments that were not targeted by SRSF1 as the positive gold standards. The length of each exonic fragment equals its counterpart in the positive gold standard sequence. We subsequently drew the same number of random sites as the predicted binding sites from each control sequence. Wilcoxon tests were also performed between predicted binding sites and the second control set of random sites. Again, all the eight nucleotidesof binding sites in CLIP sequences are significantly more single-stranded (Figure 5(B)). The boxplots of the unpaired probabilities of the random sites in the two control sets and the predicted binding sites are shown in Figure 6(A), (B) and 6(C), respectively.

The two groups of Wilcoxon testsdemonstratethat binding sites predicted by RNAMotifModeler are not only more unpaired in positive gold standard sequences than those not targeted by SRSF1, but also less structured than by chance within themselves. More interestingly, comparing Figure 5(A) with Figure 3, we found that the second and fifth nt of SRSF1 motif have much stronger sequence specificities but lower *p*-values in the Wilcoxon tests against controls, while the third, fourth, sixth and seventh positions are more significantly single-stranded but have less sequence specificities, suggesting that both the sequence and secondary structure play complementary roles in the specificity of SRSF1-RNA interactions.

### Comparisons of predictions before and after incorporating RNA structure information

**Figure 7:**
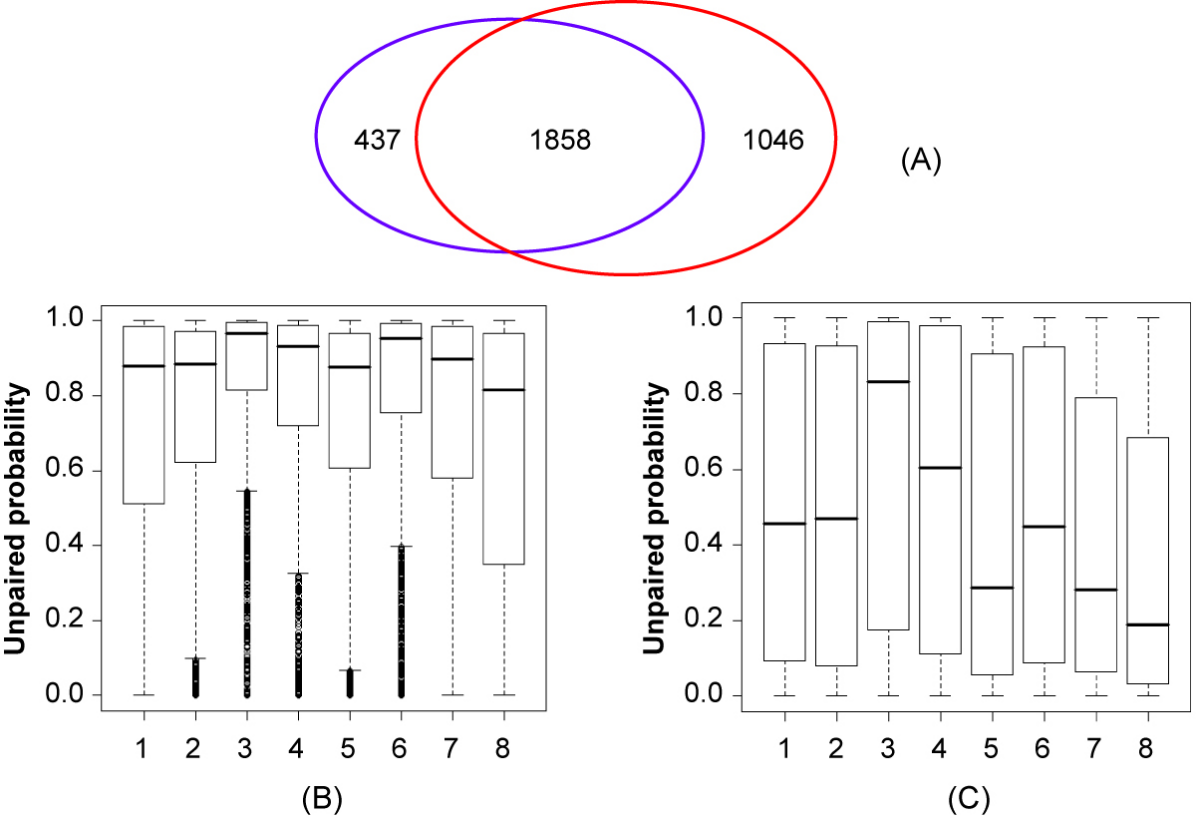
**(A) The number of binding sites predicted by RNAMotifModeler with only sequence information (blue ellipse) and incorporating structure information (red ellipse); (B) Boxplots of unpaired probabilities for 1858 binding sites both predicted by the two methods, and (C) for 437 binding sites only predicted by RNAMotifModeler without structure information**.

RNAMotifModeler can be used to predict sequence consensus motifs enriched in CLIP data without evaluating the structural context of the co-purified RNA. We then investigated whether inclusion of the unpaired probablities for each nucleotide or not contributes to the consensus motif elucidated by RNAMotifModler. Using the same positive and negative gold-standard sequences, we identified the same reference motif 'AGAAGAAG' and a very similar sequence parameter matrix. However, we obtained anAUC of0.853 and a maximal accuracy of 0.789, suggesting a slightly reduced prediction power when discarding RNA secondary structure information (Additional file 2). Using the identified parameter matrix based on only sequenceswe predicted 2295 binding sites, of which 81% are commonly identified by incorporating RNA secondary structure information (Figure 7(A)). The unpaired probabilities of the other 437 binding sites are significantly lower than previously identified binding sites (Figure 7(B) and 7(C)). Except the third nucleotide of motif, all of the unpaired probabilities of these binding sites are even lower than background, indicating that binding sites predictions may result in a considerable number of false positives caused by ignoring RNA secondary structures. Bringing in RNA secondary structure information, we found 1046 more binding sites. These binding sites may have low sequence specificities, but their binding affinities can be complemented by high structure specificities. Although the AUC increases only by 0.023 after introducing RNA secondary structure information, false positive and false negative binding sites are both significantly reduced.

## Discussion

In recent years, there is an increasing interest in using high-throughput sequencing technology to study protein-RNA binding patterns, but almost all of current bioinformatic approaches used for this purpose do not take into account RNA secondary structures, which have been demonstrated to have critical impact on protein-RNA binding in previous biochemical experiments. Thus, the starting point of our proposed model--RNAMotifModeler is to predict both structural and sequence specificities of protein-RNA binding regions. We demonstrated the potential of RNAMotifModeler by an application to predicting binding specificities of SRSF1 proteins and obtained a reference motif of 'AGAAGAAG' with a parameter matrix including retained binding affinity ratios caused by sequence degeneracy, as well as probabilities for nucleotides being unpaired.

RNAMotifModeler incorporates RNA secondary structure using RNAfold-derived probabilities of nucleotides being paired with its neighbours. The preference for base-pairing probabilities over RNA secondary structures is due to a couple of concerns: a) It is very difficult to take into account RNA secondary structures directly in many real applications because of multiple RNA folding choices including optimal and sub-optimal structures; b) Unlike MEMERIS, RNAMotifModeler tries to identify the optimal structural feature that is expected to represent the base pairing probability for each nucleotidein motif. Therefore, we did not use measurements of single-strandedness of the entire protein-RNA binding regions in MEMERIS [12]. c) The base-pairing probabilities predicted by RNAfold program [18] encode all possible secondary structures.

We note from our prediction results that almost all unpaired probabilities of bases in the reference motif of SRSF1 predicted by RNAMotifModeler are close to 1, suggesting a very strong preference of SRSF1 to single-stranded RNA. The statistical significances were further proved by Wilcoxon tests on unpaired probabilities of nucleotides between predicted binding sites and randomly selected sites in random exonic fragments that were not targeted by SRSF1. Another group of Wilcoxon tests show that the unpaired probabilities of predicted binding sites are all significantly higher than thoserandomly selected in the same positive gold-standard sequences, indicating that SRSF1 proteins indeed have strong bias to single-stranded regions. These findings are consistent with previous conclusions in the literature. It is known that SRSF1 protein contains an arginine-serine rich region (RS domain) and two RNA recognition motifs (RRMs), through which SRSF1 recognizes specific RNA regions [23,24]. Importantly, RRM is one of single-stranded RNA-binding domains of proteins [25]. Comparing the sequence consensus and p-values derived from Wilcoxon tests, we propose that sequence and structural specificities may be two complementary factors that are both facilitating the binding site recognition of SRSF1.

RNAMotifModeler also provides an option to predict only sequence consensusmotif based on gold-standard protein-RNA binding sequences. In the specific application to SRSF1, we found that the prediction power is still comparable with MEME-based approach, although the AUC and maximum accuracy were both reduced when RNA secondary structure information was not incorporated. Using predicted reference motif and sequence degeneracy parameters we identified 2295 binding sites, of which 437 are not included in the binding sites predicted after incorporating structure information. Interestingly, the unpaired probabilities of these 437 binding sites predicted by RNAfold are even lower than random sites selected by chance. Based on previous biological studies, we argue that these binding sites are probably false positives, although they satisfy the sequence specificity requirements.

Two parameters--the number of particles *M*and the contraction-expansion coefficient *β *of the Quantum Particle Swarm Optimization greatly affect the predicting accuracy of RNAMotifModeler. To estimate and set up these parameters prior to the optimization procedure, we did a series of hexamer motif searching tests with *M*enumerated from 10 to 10000 and *β *ranging from 0 to 1 for SRSF1 CLIP-seq data. The AUC scores resulted from optimizations using all parameter combinations are presented in 3D heatmaps (Additional file 3). We observed a much more rapid decrease in prediction power as *β *becomes lower when *M *is small. In contrast, when *β *is sufficiently high, the AUC score is not greatly affected by *M*. Thus, the greater *M *and *β *are, the higher prediction performance RNAMotifModeler would achieve. However, under the consideration of computational efficiency, we have to consider the time consumed in each test (Additional file 3). The time consumed is exponential to the increment of the number of particles, and is not actually controlled by *β*. When *m *is 100 and *β *equals 1.0, RNAMotifModeler achieved a high AUC score of 0.86 within three minutes. These two parameters are then selected for all the other optimizations for the SRSF1 dataset used in this study.

Convergence of optimization algorithms used in predicting protein-DNA or protein-RNA binding sites is a common concern due to a number of parameters to be fitted in model. In this report, we proposed motif searching pathways and motif searching graphs to inspect whether or not the algorithm of RNAMotifModeler indeed has a good convergence property. Importantly, the convergence of randomly initialized motif candidate to final targets turned out to be very fast. Thus, for short motifs, we suggest generate such a motif searching graph in order to have a global landscape of all possible converged motifs and their possible relationships, as they are possibly parts of a common longer motif.

Despite our successful characterization of the binding features of SRSF1 proteins, our future work will be applying RNAMotifModeler to studying specificities of other RNA binding proteins such as fox2, NOVA and EWS, for which high-throughput sequences are available.

## Methods

### Predicting RNA base-pairing probabilities

One of the distinct features of RNAMotifModeler is that the information of secondary structures of the RNA regions bound by SRSF1 proteins is incorporated into the motif identification. For each nucleotide in the RNA fragment, we calculate the base pairing probability using the RNAfold function of the Vienna RNA package (version 1.7.1) [18]. The base pairing probability is used since it integrates likelihood of single-strandedness over multiple possible RNA secondary structures. For the CLIP-seq derived RNA fragments, these probabilities are generated based on the base pairing probability of base *i *being paired with base *j*, denoted as *p_i,j_*. The binding probability of base *i *with all other neighbouring bases, defined as *P_i_*, is calculated by:

(1) $$P_{i}=\overset{n_{s}}{\underset{j=i+1}{\sum }}P_{ij}+\overset{i-1}{\underset{j=1}{\sum }}P_{ji,}$$

where *n_s _*is the length of sequence *s*. Similar strategies are also used elsewhere [26,27].

### Modelling protein-RNA binding affinities

In *RNAMotifModeler*, the consensus of each binding motif is defined by the following components: 1) The reference motif, a *k*-base RNA sequence on which the protein preferably binds; 2) Retained binding affinity despite of a one-nucleotide deviation from reference motif to the sequence of one binding sites. For each *k*-base motif, there are *3k *retained binding affinities that describe all the possible deviations from reference motif. For instance, if the *i*-th base of the reference motif and a specific binding site is *m_i _*and *f_i_*, respectively, the retained binding affinity is defined as $\mu_{i,m_{i},f_{i}}$; 3) a vector that denotes the optimal base pairing probability of *k *bases in the motif **θ **= (*θ_i_*); and 4) the penalty for the deviation from the optimal base pairing probability *α*. All these parameters will be optimized iteratively. A matching score describing the similarity between an RNA fragment (*F*) and a reference motif (*R*) is defined:

(2) $$S_{R,F}=\overset{L-k+1}{\underset{l=1}{\max}}\left(S_{R,F,l}\right),$$

Where *S*_*R*,*F*,*l *_is the binding affinity for *l*-th binding site:

(3) $$S_{R,F,l}=\overset{k}{\underset{i=1}{\prod }}\left(\left(\mu_{i,m_{i},f_{l,i}}\right)\left(1-\alpha ∙\left|\theta_{i}-P_{f_{l,i}}\right|\right)\right),$$

where $P_{f_{i}}$ represents the pairing probability of the *i*-th nucleotide in the RNA fragment *F*, calculated in Eq. (1). This matching score integrates the loss of binding affinity caused by both nucleotide and structure deviances from reference motif. We denote the parameter associated to the reference motif *R *as **λ*_R _***= **(μ,θ**, *α*)***_R_***, where **μ,θ **and *α*represent the *3k *retained binding affinities, optimal base pairing probability of *k *bases, and the penalty for the deviation from the optimal base pairing probability, respectively.

#### Identify the optimal reference motif from CLIP-seq data

We adopted an iterative approach to identify the optimal reference motif and its associated parameters, using a Quantum Particle Swarm Optimization algorithm (QPSO) [28]. The iterative strategy includes the selection of reference motif*R*, and optimization of the parameters associated to the reference motif **λ*_R_***. The overall procedure includes the following steps:

1. Randomly select a motif candidate *R_init _*from the motif searching space **M **= {*b*_1_*b*_2_...*b_k _*: *b*_1_,*b*_2_,...*b_k _*∈ {A,G,C,U}}as the reference motif.

2. Optimize the parameters for the reference motif by maximizing its ability for characterizing the CLIP-seq-derived RNA fragments.

Step 2.1. Parameter initiation. We first create M particles in the parameter space by randomly selecting numbers from U(0,1).

Step 2.2. Particle evaluation. For each particle (parameters), we evaluate its capability for distinguishing the CLIP-seq-derived RNA fragment from background sequences. We plot an ROC (Receiver Operating Characteristic) curve by adjusting the matching score threshold, calculated in Eq. (2). The quality of the parameter is evaluated based on the AUC (area under the curve) of the ROC plot.

Step 2.3. Particle update. Let $\lambda_{i}^{selfbest}\left(t\right)$ and ***λ**^globalbest^*(*t*) be the best individual particle and the population of particles has met at the *t*-th iteration. To guarantee convergence, each particle must converge to its local attractor $\lambda_{i}^{pbest}$[28]. Compute $\lambda_{i}^{pbest}\left(t\right)$ and the mean of the best positions of all particles $\lambda^{mbest}\left(t\right)$ as follows:

(4) $$\lambda_{i,j}^{pbest}\left(t\right)=\left(\varphi_{1}\cdot \lambda_{i,j}^{selfbest}\left(t\right)+\varphi_{2}\cdot \lambda_{j}^{globalbest}\left(t\right)\right)∕\left(\varphi_{1}+\varphi_{2}\right)$$

(5) $$\lambda_{j}^{mbest}\left(t\right)=\overset{m}{\underset{i=1}{\sum }}\lambda_{i,j}^{pbest}\left(t\right)∕m,$$

where *φ_1 _*and *φ_2_*are random variables following U(0,1);

QPSO employs Monte Carlo method to update parameters:

(6) $$\lambda_{i,j}\left(t+1\right)=\left\{\begin{array}{c} \lambda_{i,j}^{pbest}\left(t\right)-\beta \cdot |\lambda_{j}^{mbest}\left(t\right)-\lambda_{i,j}\left(t\right)|\cdot \ln\left(1∕u\right),\;k\geq 0.5\\ \lambda_{i,j}^{pbest}\left(t\right)+\beta \cdot |\lambda_{j}^{mbest}\left(t\right)-\lambda_{i,j}\left(t\right)|\cdot \ln\left(1∕u\right),\;k<0.5 \end{array},\right)$$

where *β *is called contraction-expansion coefficient controlling the convergence speed of QPSO; *u *and *k *are random variables which also follow U(0,1).

Repeat Step 2 and Step 3 until |***λ**^globalbest^*(*t*+1)- ***λ**^globalbest^*(*t*)| < ε repeatedly, in which *ε *is a tolerance used here as a criterion for the algorithm to terminate;

3. Based on the final parameter vector ***λ**^globalbest^*, the maximal binding affinity of motif candidate *K *in positive gold standard sequence *F *is:

(7) $$a_{K,F}=\underset{\sigma \in \Omega_{K,F}}{\text{Max}}a_{K,F,\sigma },$$

where *Ω_K,F _*denotes the set of all binding sites for motif *K *in sequence *F*; *a_K,F,σ _*is also computed by Eq. (3).

Let *n_s _*and *n_m _*be the number positive gold standard sequences and the number of motif candidates, respectively. Let $S_{R_{init},F}$ be the maximal binding affinity computed using optimized parameters for the initial reference motif *R_init _*in sequence *F*. Although *R_init _*is a reference motif, $S_{R_{init},F}$ is not necessarily contributed by a binding site instance of *R_init_*. In contrast, the 'real' reference motif contributes are always expected to contribute more to the binding affinities. Thus, to evaluate contributions of all motif candidatesto binding affinities ofpositive gold standard sequence, we define $c={\left[c_{F,K}\right]}_{F=1,2,...,n_{s},K=1,2,...,n_{m}}$ as the motif contribution scorematrix:

(8) $$c_{F,K}=\left\{\begin{array}{c} 0,\;a_{K,F}\neq S_{R_{init},F}\\ 1,\;a_{K,F}=S_{R_{init},F} \end{array}\right),$$

and $v={\left[v_{K}\right]}_{K=1,2,...,n_{m}}$ as the motif contribution score vector:

(9) $$v_{K}=\overset{n_{s}}{\underset{F=1}{\sum }}c_{F,K},$$

We denote the motif associated with the maximum score in **v **as *R_max_*. If *R_max _*= *R_init_*, meaning the initialized reference motif accounts for the most contribution to the retained binding affinities, then we stop the iteration; otherwise, let *R_max _*be the next *R_init_*, and repeat steps 2 and 3 until convergence.

### Motif searching pathways and graph

The route from original assumed reference motif to the final converged motif is called a *motif searching pathway*. Different initialized motifs may converge to different final motifs. Therefore, to investigate the convergence performance of RNAMotifModeler, it is important to enumerate all possible convergence pathways and find out what are the final converging points. For the specific example of SRSF1 protein, we used RNAMotifModeler to predict the optimal parameters for each one of 4096 motif candidates. All pathways are summarized and illustrated in a graph in Figure 2 using Cytoscape (version 2.6.1) [29]. Source motifs (all initial motifs), intermediary motifs (motifs which are neither final nor initial motifs) and destination motifs (converged motifs) are colored in blue, purple and red colors, respectively. The arrow from a source motif to an intermediary or destination motif denotes one motif transit. From the graph, we observe that the vast majority of original motifs transited to only three motifs, which we believe are the best candidates of reference motifs for SRSF1 proteins.

### RBP binding motif logo

Although RNAMotifModeler provides a parameter matrix consisting of retained binding affinity ratios due to sequence mutations and structure alterations at each base, it is not straightforward for people to comprehend the sequence consensus. Thus, we provide an alternative way to generate a Positional Weight Matrix (PWM) and corresponding sequence logo. First of all, once RNAMotifModeler reaches a convergence, we obtain the optimal reference motif, estimated parameters, ROC curve, AUC score and the accuracy curve. At the peak of the accuracy curve we choosecorresponding binding affinity as a cutoff. Then, we trace back to each positive gold standard sequence and search all binding sites with binding affinities higher than the cutoff score. These predicted binding sitesare subsequently used to compute a corresponding PWM and create a sequence logo based on Weblogo [19].

## Competing interests

The authors declare that they have no competing interests.

## Authors' contributions

XW and YL contributed to the design of the study. XW and YL designed and performed the computational modelling and drafted the manuscript. JRS provided the CLIP-seq data for SRSF1 proteins. XW, LJ, JRS, JL, KW and YL participated in coordination, discussions related to result interpretation and revision of the manuscript. All the authors read and approved the final manuscript.

## Supplementary Material

Additional file 1 **Optimal 6nt and 7nt sequence-structural consensus for SRSF1 proteins predicted by RNAMotifModeler**. The upper panel (A) and the lower panel (B) show the sequence and structural parameters identified for motif of length 6nt and 7nt, respectively. Click here for file

Additional file 2 **Prediction results based on RNAMotifModeler excluding the information of RNA secondary structure**. (A) ROC curve (B) Accuracy curve, and (C) consensus sequence logo. Click here for file

Additional file 3 **3D heatmaps illustrating (A) the prediction power and (B) time cost of RNAMotifModeler affected by the number of particles and the Contraction-Expansion coefficient which are two critical parameters of QPSO algorithm**. Click here for file
