# Supplementary material for: Predicting sequence and structural specificities of RNA binding regions recognized by splicing factor SRSF1
Source: BMC Genomics. 2011 Dec 23;12(Suppl 5):S8. doi: 10.1186/1471-2164-12-S5-S8 (PMC3287504; doi:10.1186/1471-2164-12-S5-S8)
Supplement: Additional file 1 — Optimal 6nt and 7nt sequence-structural consensus for SRSF1 proteins predicted by RNAMotifModeler. The upper panel (A) and the lower panel (B) show the sequence and structural parameters identified for motif of length 6nt and 7nt, respectively. [file 1471-2164-12-S5-S8-S1.pdf]

(A)

	G	A	A	G	A	A
**A**	0.32	1.00	1.00	0.08	1.00	1.00
**G**	1.00	0.69	0.87	1.00	0.79	1.00
**C**	0.08	0.33	0.13	0.41	0.42	0.68
**U**	0.05	0.23	0.71	0.07	0.24	0.94
**UP**	0.96	0.99	0.61	0.89	0.98	0.79

(B)

	A	G	A	A	G	A	A
**A**	1.00	0.14	1.00	1.00	0.26	1.00	1.00
**G**	0.96	1.00	0.49	0.97	1.00	0.82	0.99
**C**	0.72	0.22	0.46	0.12	0.41	0.32	0.67
**U**	0.92	0.08	0.40	0.73	0.07	0.00	0.72
**UP**	1.00	0.94	1.00	0.97	0.91	0.99	0.75
