# Supplementary figures and images for: Predicting sequence and structural specificities of RNA binding regions recognized by splicing factor SRSF1
Source: BMC Genomics. 2011 Dec 23;12(Suppl 5):S8. doi: 10.1186/1471-2164-12-S5-S8 (PMC3287504; doi:10.1186/1471-2164-12-S5-S8)

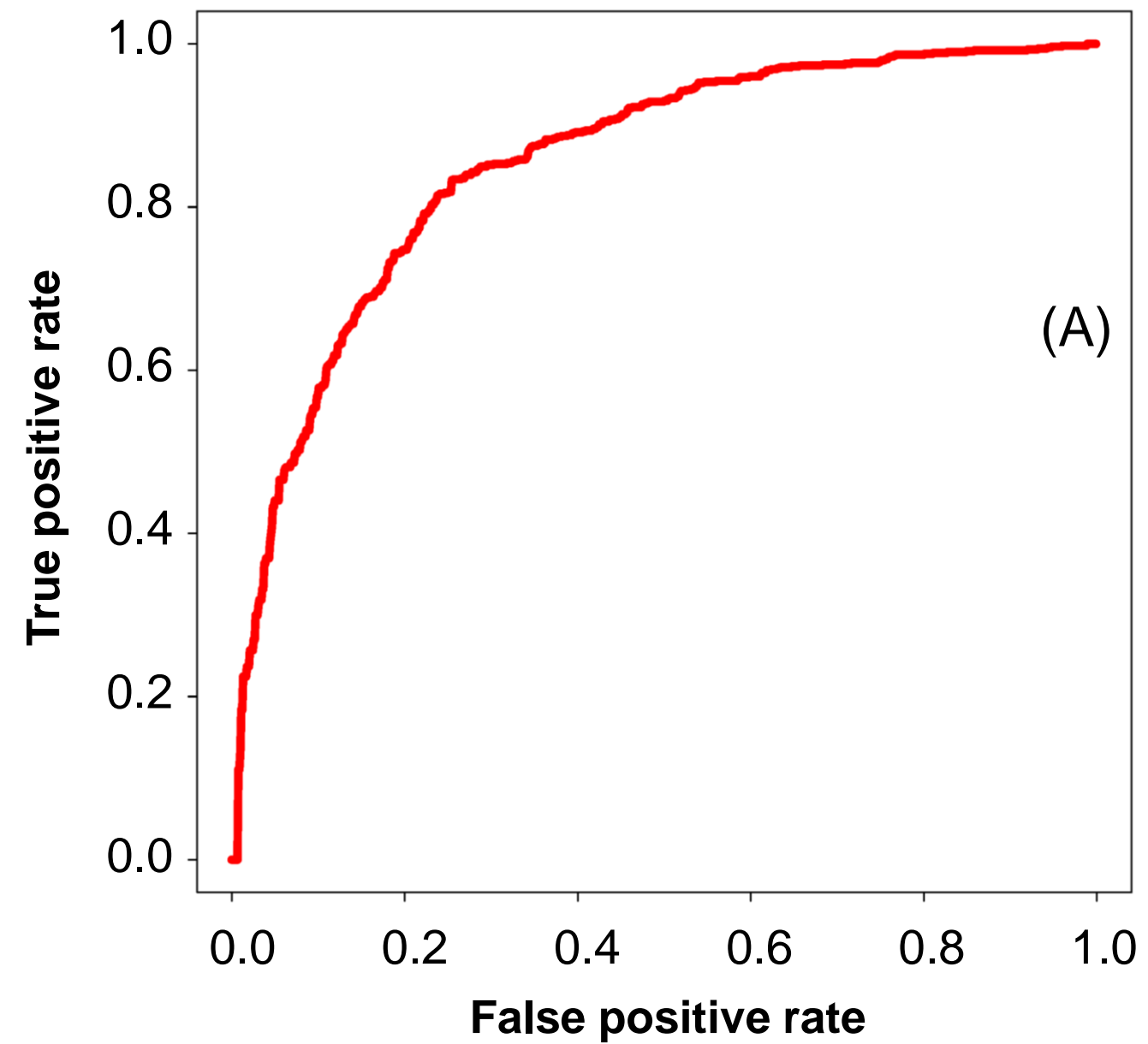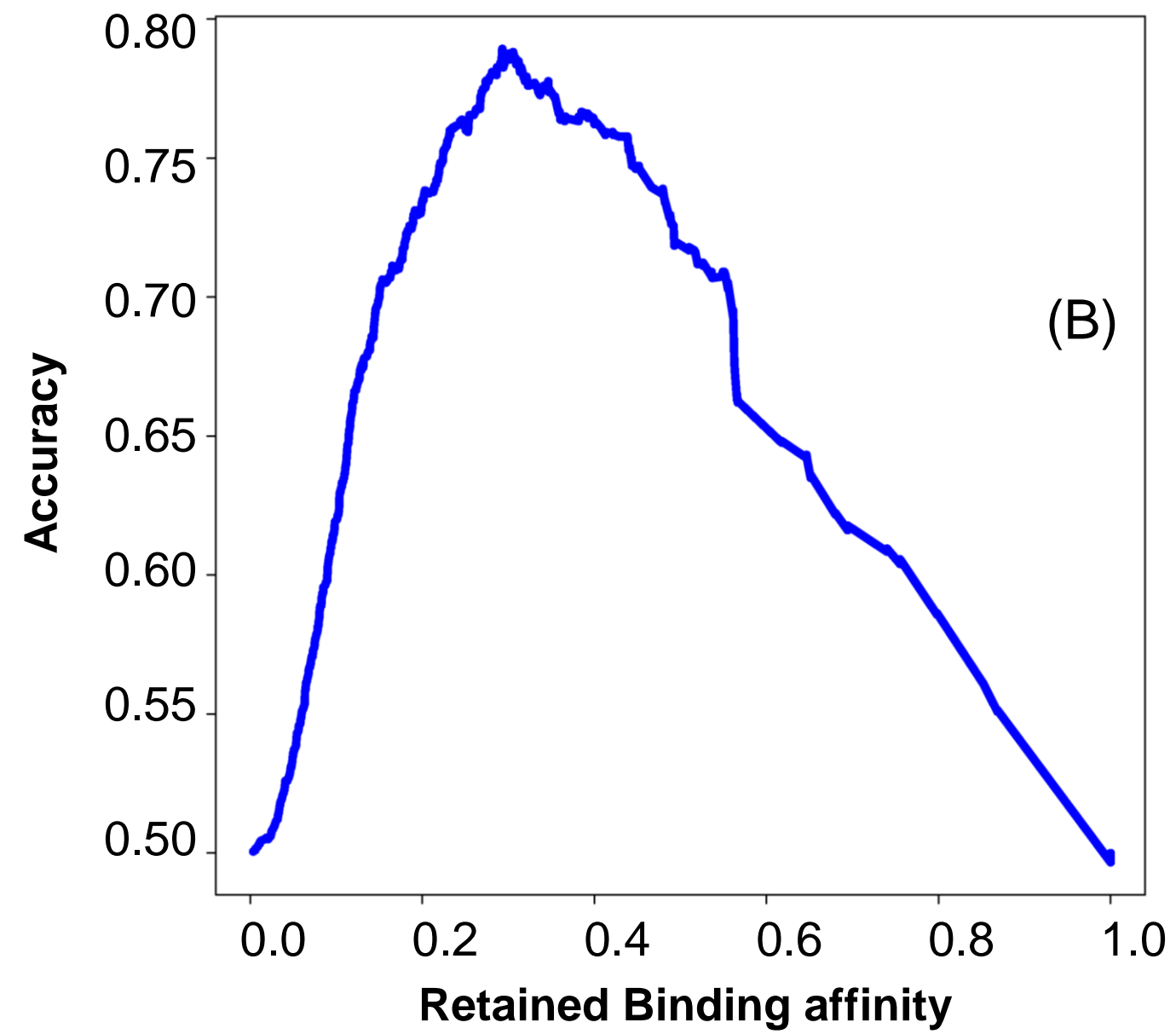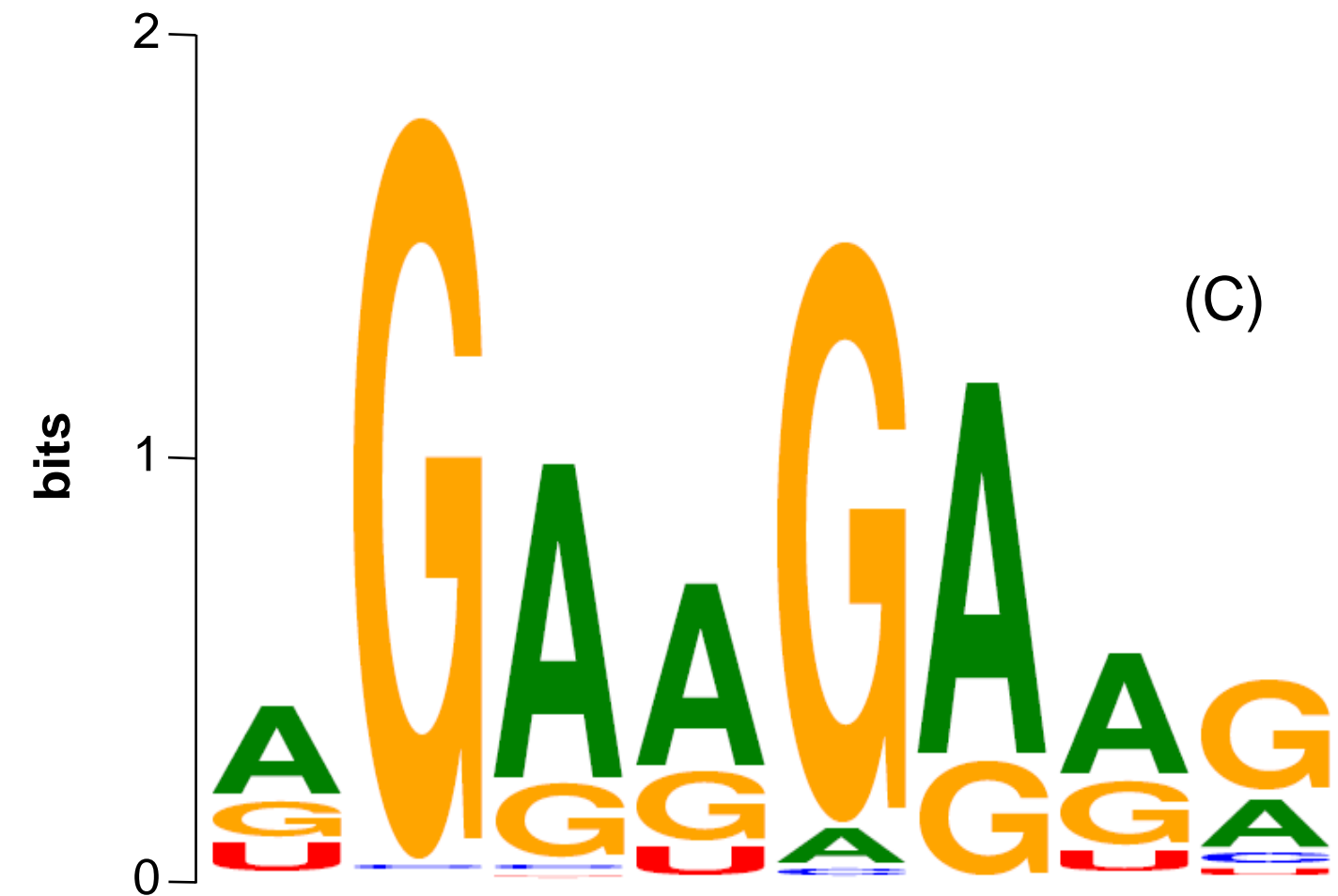

Supplement: Additional file 2 — Prediction results based on RNAMotifModeler excluding the information of RNA secondary structure. (A) ROC curve (B) Accuracy curve, and (C) consensus sequence logo. [file 1471-2164-12-S5-S8-S2.pdf]

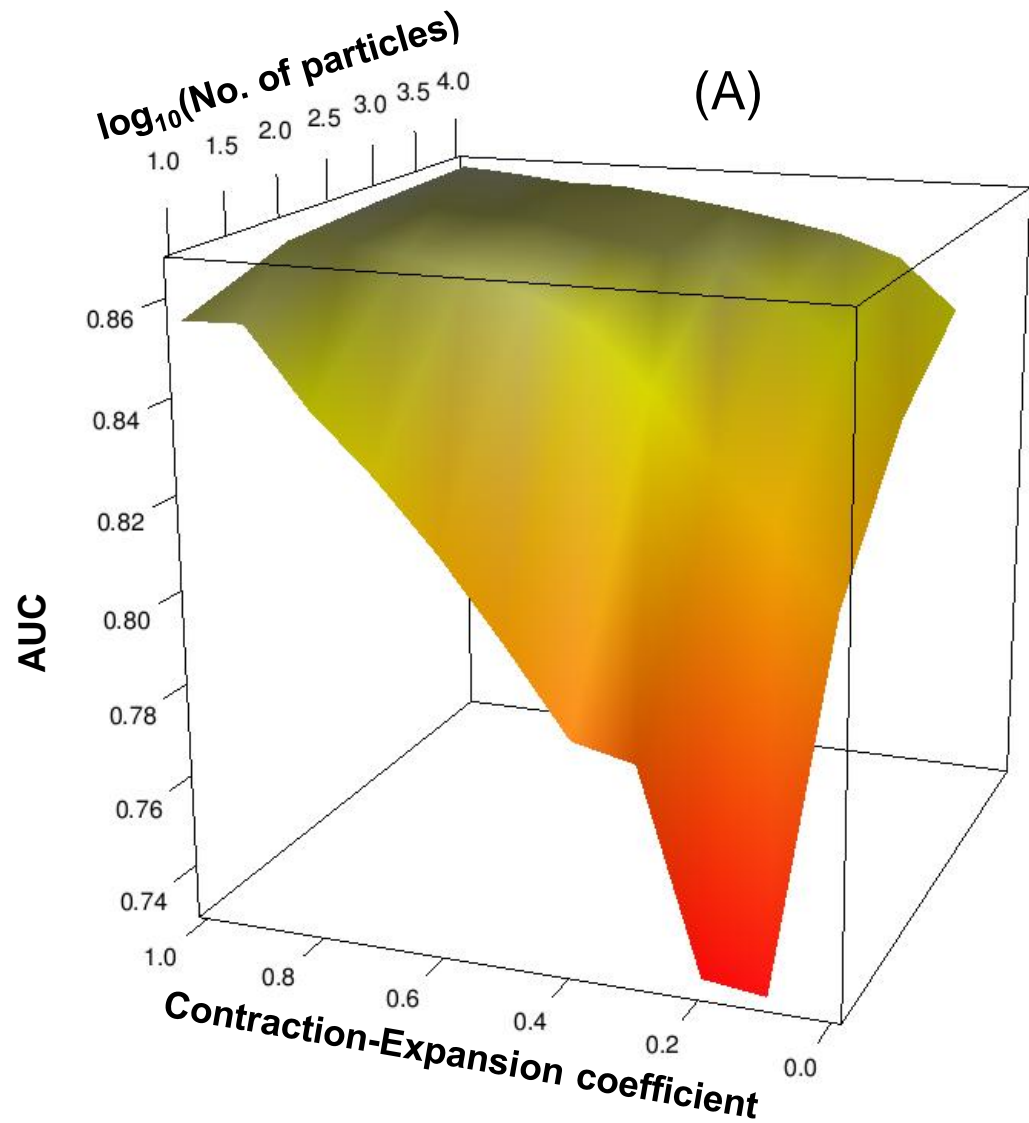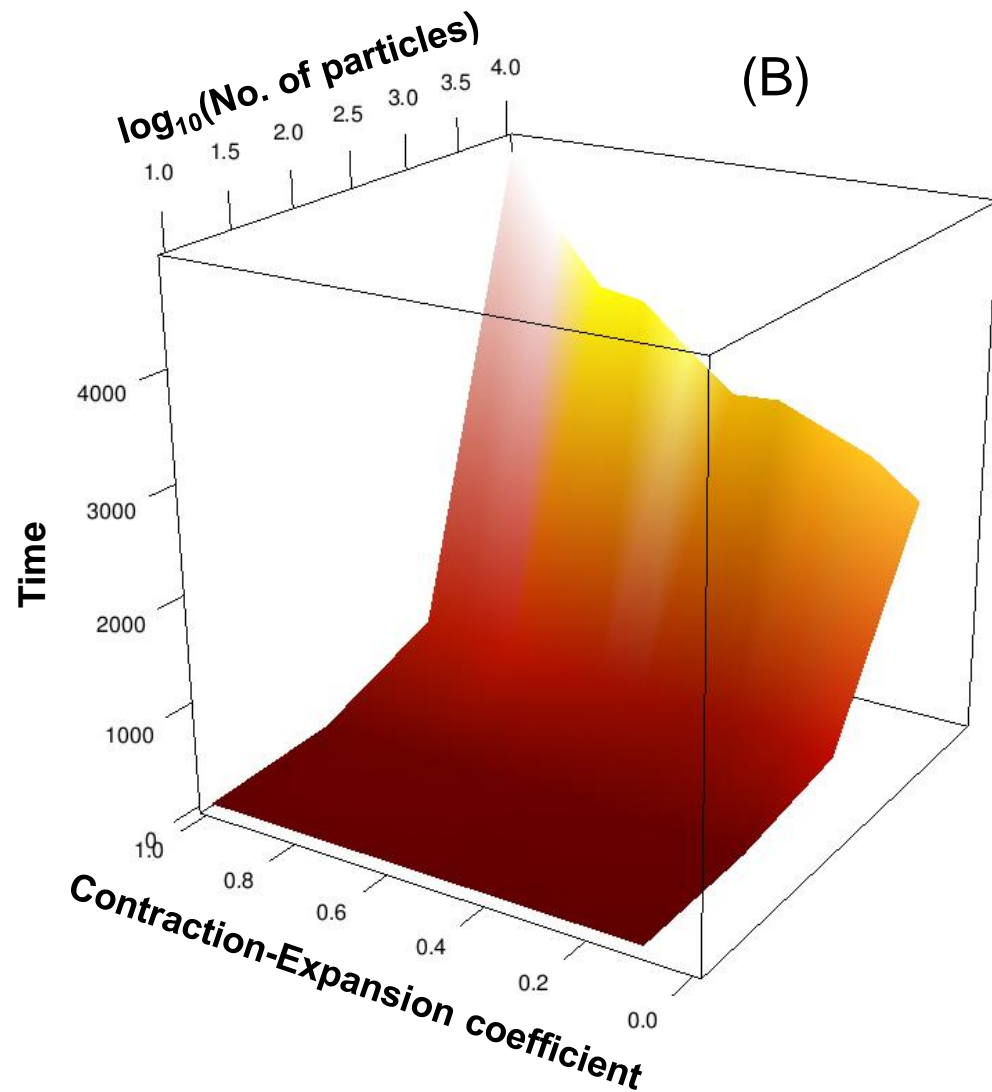

Supplement: Additional file 3 — 3D heatmaps illustrating (A) the prediction power and (B) time cost of RNAMotifModeler affected by the number of particles and the Contraction-Expansion coefficient which are two critical parameters of QPSO algorithm. [file 1471-2164-12-S5-S8-S3.pdf]
